# Mate availability does not influence mating strategies in males of the sexually cannibalistic spider *Argiope bruennichi*

**DOI:** 10.7717/peerj.5360

**Published:** 2018-08-07

**Authors:** Anna-Lena Cory, Jutta M. Schneider

**Affiliations:** Institute of Zoology, Universität Hamburg, Hamburg, Germany

**Keywords:** Female density, Male mating effort, Male sacrifice, Sex pheromones, Sexual cannibalism

## Abstract

**Background:**

Sexual selection theory predicts that male investment in a current female should be a function of female density and male competition. While many studies have focused on male competition, the impact of female density on male mating investment has been widely neglected. Here, we aimed to close this gap and tested effects of mate density on male mating decisions in the orb-web spider *Argiope bruennichi*. Males of this species mutilate their genitalia during copulation, which reduces sperm competition and limits their mating rate to a maximum of two females (bigyny). The mating rate is frequently further reduced by female aggression and cannibalization. Males can reduce the risk of cannibalism if they jump off the female in time, but will then transfer fewer sperm. An alternative solution of this trade-off is to copulate longer, commit self-sacrifice and secure higher minimal paternity. The self-sacrificial strategy may be adaptive if prospective mating chances are uncertain. In *A. bruennichi*, this uncertainty may arise from quick changes in population dynamics. Therefore, we expected that males would immediately respond to information about low or high mate availability and opt for self-sacrifice after a single copulation under low mate availability. If male survival depends on information about prospective mating chances, we further predicted that under high mate availability, we would find a higher rate of males that leave the first mating partner to follow a bigynous mating strategy.

**Method:**

We used naïve males and compared their mating decisions among two treatments that differed in the number of signalling females. In the high mate availability treatment, males perceived pheromone signals from four adult, virgin females, while in the low mate availability treatment only one of four females was adult and virgin and the other three were penultimate and unreceptive.

**Results:**

Males took more time to start mate searching if mate availability was low. However, a self-sacrificial strategy was not more likely under low mate availability. We found no effects of treatment on the duration of copulation, the probability to survive the first copulation or the probability of bigyny. Interestingly, survival chances depended on male size and were higher in small males.

**Discussion:**

Our results do not support the hypothesis that mate density variation affects male mating investment, although they clearly perceived mate density, which they presumably assessed by pheromone quantity. One reason for the absence of male adjustments to mating tactics could be that adaptations to survive female attacks veil adaptations that facilitate mating decisions.

## Introduction

The mating decisions of males often reflect the general trade-off between increasing either mating rates or the investment in the current mate (Magrath & Komdeur, 2003; Scharf, Peter & Martin, 2013; Trivers, 1972). The trade-off results from the risk of losing paternity to rival males, since females are often choosy and polyandrous (Arnqvist & Nilsson, 2000; Jennions & Petrie, 1997; Jennions & Petrie, 2000; Parker, 1970). Strategies that secure or optimize paternity gains by increasing their reproductive effort are mate guarding, nuptial feeding, mate plugging, courtship behaviour, male fighting, and ejaculate expenditure (Alatalo, Höglund & Lundberg, 1991; Andersson, 1994; Eberhard, 1994; Uhl, Nessler & Schneider, 2010; Vahed, 1998; Wedell, Gage & Parker, 2002). However, increased reproductive effort has costs such as the depletion of resources (e.g., the amount of sperm), or the consumption of time and energy (Cordts & Partridge, 1996; Hughes et al., 2000; Saeki, Kruse & Switzer, 2005; Sparkes, Keogh & Pary, 1996). Accordingly, males that allocate more time, energy or resources to one mating partner, risk depleting reserves for future mates and missing further mating opportunities. Hence, the decision whether males should invest in the current or future mating partners should strongly depend on mating opportunities (Kokko & Rankin, 2006; Kvarnemo & Ahnesjo, 1996).

Mating opportunities for males are a function of female density and male competition (Kvarnemo & Ahnesjo, 1996), although many studies focus on the latter. There is ample evidence that males invest more in mate guarding, nuptial gifts, and/or ejaculates in response to a strong risk of male competition (Bailey, Gray & Zuk, 2010; Carroll & Corneli, 1995; DelBarco-Trillo, 2011; Weir, Grant & Hutchings, 2011). In contrast, the effects of female density on male mating investment are rarely investigated, and if considered they are often veiled in studies regarding population densities or sex ratios (Barrett, Evans & Gasparini, 2014; Gage, 1995; Kokko & Rankin, 2006; Lauer, Sih & Krupa, 1996; Linklater et al., 2007; Oku, 2009). Studies about sex ratios indeed depict the actual mating opportunities but disregard that under natural conditions, gathering information about mating opportunities may be constrained by the male’s inability to perceive social cues. For instance, in mating systems where males are adapted to locate females by female sex pheromones or sex-specific chemical cues (Wyatt, 2014), males may not necessarily perceive sex-specific chemical cues of conspecific males. In this scenario, males are not able to determine the current sex ratio but can assess female density with some accuracy.

Female localization through chemical cues (or sex pheromones) is particularly common in moths, mantises, and spiders (Gaskett, 2007; Greenfield, 1981; Holwell, Barry & Herberstein, 2007; Maxwell, Barry & Johns, 2010; Schulz, 2013). Some members of these taxa are known to assess female traits such as body condition or mating status by using chemical cues (e.g., Barry, 2010; Foster & Johnson, 2011; Stoltz, McNeil & Andrade, 2007). Furthermore, in several spider species males developed faster if they received volatile or silk-based pheromonal cues produced by females (Cory & Schneider, 2018; Kasumovic & Andrade, 2006; Neumann & Schneider, 2016). These findings imply that chemical cues are also involved in the assessment of female availability. Other studies suggest that males adjust their mating investment in response to experience with chemical cues produced by females. For example, it was found that males of the cellar spider *Pholcus phalangioides* courted longer if they had experienced silk cues of non-virgin, but receptive females (Hoefler, 2007). However, it is unknown whether males also adjust their mating investment in the current mate immediately on the receipt of chemical information about female availability. Immediate responses to social information may be particularly relevant in species where the availability of mating partners can be very dynamic throughout the mating season, as is the case in some well-studied orb-web spiders (Foellmer & Fairbairn, 2005; Zimmer, Welke & Schneider, 2012).

In many spiders, males show an extremely high limitation in mating rates because, with each mating encounter, males risk being cannibalized by the female (Elgar & Crespi, 1992). For instance, in the orb-web spider *Argiope bruennichi*, 50–80% of males die after a single copulation (Fromhage, Uhl & Schneider, 2003; Welke, Zimmer & Schneider, 2012). The causes of sexual cannibalism are still debated and are diverse (Elgar & Schneider, 2004; Schneider, 2014; Wilder, Rypstra & Elgar, 2009). Some studies have found that sexual cannibalism depends on the hunger state of females and may be an adaptation to food limitation in nature (Berning et al., 2012; Moskalik & Uetz, 2011). Apart from ecological reasons, sexual cannibalism may also be considered a manifestation of sexual conflict (Schneider, 2014). While some studies suggest that sexual cannibalism give females control over mating (Anderson & Hebets, 2017; Schneider et al., 2006), other studies indicate that males actively sacrifice their life (Andrade, 1996; Schneider et al., 2006; Schwartz, Wagner & Hebets, 2013). A male benefits from self-sacrifice if his body contributes to the female’s fecundity or offspring survival and if paternity security is high (Elgar & Nash, 1988; Welke & Schneider, 2012). However, as with any reproductive effort, self-sacrifice has to be traded off against future mating opportunities and should, therefore, depend on female availability (Fromhage & Jennions, 2016).

Extreme male mating investment including self-sacrifice has evolved independently in some spider taxa limiting males to maximally two mating chances (Schneider & Fromhage, 2010). The limitation arises because their paired genitalia (pedipalps) also function as mating-plugs in the equally paired female genital openings (Uhl, Nessler & Schneider, 2010). Mate plugging by genital mutilation may indeed prevent insemination by rivals but limits males to a single use of each pedipalp (Uhl, Nessler & Schneider, 2010). Accordingly, males can only invest in one (monogyny) or two females (bigyny). A mating system, in which bigyny and male sacrifice behaviour coexist, provides a good opportunity to test effects of female availability because poor mating decisions have severe fitness consequences.

To test predictions about the density-dependent reproductive investment of males, we used the highly cannibalistic *A. bruennichi*, in which males show monogynous and bigynous mating tactics (Fromhage, Uhl & Schneider, 2003; Welke, Zimmer & Schneider, 2012). Females stereotypically attack males during copulation, but males can reduce the risk of cannibalization if they copulate briefly and try to jump off in time (Fromhage, Uhl & Schneider, 2003; Schneider, Fromhage & Uhl, 2005; Welke & Schneider, 2010). By decreasing copulation duration, males also reduce the amount of sperm they transfer (Schneider et al., 2006). The trade-off between sperm transfer and mortality risk is further complicated because a considerable risk of mortality remains even after a short copulation (Fromhage, Uhl & Schneider, 2003; Welke & Schneider, 2010). Accordingly, self-sacrifice after a long copulation (more than 10 s) can be considered a comparably low-risk strategy because the outcome is predictable and 50% of the life-time sperm load can be transferred (Fromhage, Uhl & Schneider, 2003; Welke & Schneider, 2010). On the other hand, surviving males have the chance to either minimize the risk of sperm competition in the first female by plugging the second genital opening of the same female or to continue mate searching (bigyny) (Nessler, Uhl & Schneider, 2007; Welke, Zimmer & Schneider, 2012). A successful bigynous strategy can enhance the overall fitness of a male, but a single copulation leaves one female genital opening unsecured (Nessler, Uhl & Schneider, 2007; Welke, Zimmer & Schneider, 2012). Moreover, bigynous males also need to find another mating partner, which should strongly depend on female availability (Fromhage & Schneider, 2012). Thus, under a low density of females, it may be better to increase the investment in the current mating partner particularly if male competition is high. Strong male competition is generally expected in such mating systems because they are associated with extreme female-biased sexual size dimorphism (females more than twice as large as males; Hormiga, Scharff & Coddington, 2000; Scharff & Coddington, 1997) and reduced mortality of males during development, leading to a male-biased sex ratio (Danielson-Francois et al., 2012; Foellmer & Fairbairn, 2005; Fromhage, Elgar & Schneider, 2005; Fromhage & Schneider, 2005).

For our experiments, we used males that had no experience with female cues until we tested their mating decisions in response to different levels of female density. To manipulate information about female density, we made use of the fact that in *A. bruennichi* only adult, virgin females produce a sex pheromone that attracts males (Chinta et al., 2010). We conducted mating tests in which males were exposed to either four adult, virgin females (high mate availability) or one adult, virgin and three penultimate females (low mate availability) that do not produce the sex pheromone. We chose the term “mate availability” instead of “female density” because we used the same total female density for both treatments. We predicted that under low mate availability, copulation duration and the likelihood of sexual cannibalism would be higher than under high mate availability. Since our predictions are based on the assumption that males adjust their mating investment to the chance of finding prospective mates, we also predicted that if mate availability and thus prospective mating chances are low, males would rather stay with the current mate than continue mate searching.

## Material and Methods

### Spider husbandry

We used laboratory-bred *A. bruennichi* that derived from 37 egg sacs and whose parents originated from Southern France (Cory & Schneider, 2018). All spiders were kept in cups that were turned upside down and had a hole filled with cotton wool. Also, the cups were roughened from the inside to facilitate web-building inside the cups. Cup volume (from 50 ml to 1 l) and food provision was adjusted to the spiders’ body size. Small spiders received pollen and approximately three dead *Drosophila melanogaster* three times a week. Two days a week, larger individuals received ca. 20 *Drosophila* spp*.* and large subadult and adult females were fed with three *Calliphora* sp. Spiders were provisioned with water six times a week. Generally, we sprayed water inside the cups; but on days after food provision, we only watered the cotton wool to prevent the prey from escaping. During water provision, we also checked for moults of penultimate males and females and registered the day of maturation. Penultimate and adult spiders can be recognized and distinguished by their genitalia. Males develop a genital bulb on the distal part of each of their pedipalps (Uhl, Nessler & Schneider, 2007). The genital bulb is undifferentiated in penultimate males whereas it shows discrete sclerotized structures in adult males. Females can be identified by the scape, which is a sclerotized structure covering the epigyne (Uhl, Nessler & Schneider, 2007). The scape already starts to develop during the penultimate stage but is shorter and less differentiated than in adult females. Furthermore, the scape of penultimate females is covered by a thin membrane and is only exposed after females moult to sexual maturity. Females of both maturation states were put in frames (trapezoidal shape: upper width 14.5 cm, lower width 9 cm, length 13 cm) that could be fixed in our test arenas (see experimental design), which offered males access to the females during the mating tests.

Spiders either died a natural death in the laboratory and/or were frozen by −80° C. Some males were sexually cannibalized during the mating tests, which conforms to the natural situation. We used the tibia-patella length as an index for body size. To measure leg length, we photographed the legs under a binocular and used the measuring tools in the software Leica Application Suite V4.6 (Leica Microsystems, Heerbrugg, Switzerland). In addition to body size, which is fixed after maturation, we also determined the weight shortly after maturation and on the test day with a calibrated scale (Mettler Toledo AB54-S; accuracy 0.1 mg).

### Treatment groups

We tested mating decisions of naïve males with no prior experience with female cues in two experimental set-ups with either a low or high availability of mating partners. In both treatments, males were exposed to four females, but we manipulated the number of pheromone-emitting females.

The low mate availability treatment was composed of one adult, virgin and three penultimate females. We know from previous studies in *A. bruennichi* that adult, virgin females are highly receptive whereas penultimate females are not (Schneider et al., 2006, A Cory, pers. obs., 2014). Furthermore, subadult females do not produce the sex pheromone that was found on the body and silk of adult, virgin females and that attracted males (Chinta et al., 2010).

In the high mate availability treatment all females were adult and virgin. The four females in each high mate availability treatment were matched for adult age (we only allowed an age difference of two days) because previous studies found that mate attraction depends on female age (Cory & Schneider, 2016). We ensured that the mean ages of the four females in the high mate availability treatment did not differ from the ages of the adult, virgin females in the low mate availability treatment (Mann Whitney *U* test: *N* = 50, *W* = 288, *p* = 0.8167).

**Figure 1:**
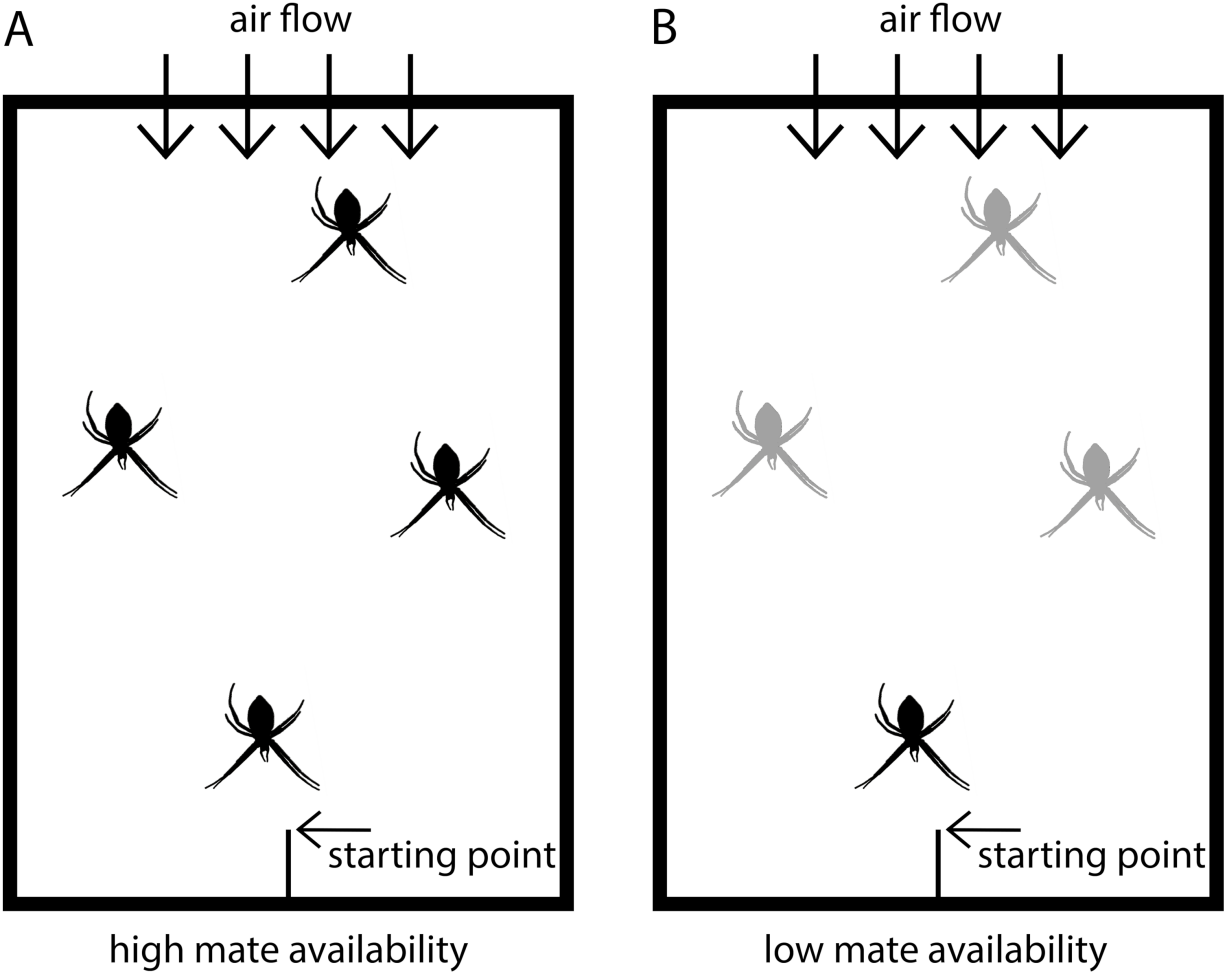
Scheme of experimental set-up. Males were placed in test arenas (100 × 70 × 50 cm) and exposed either to (A) four virgin adult females (high mate availability) or to (B) one adult, virgin female and three penultimate females (low mate availability). Adult virgin females are black and penultimate females grey-colored.

While we controlled for the adult age, we were unable to match for body size, which is a common measure of female quality. To minimize potential effects of female quality, we used individuals from Southern France for which previous studies found that female body size had no effect on male mating decisions (Cory & Schneider, 2018). Males generally mated with the first female they encountered. Body sizes of the females that copulated in both treatments revealed no difference (*t* test: *N* = 50, *df* = 48, *t* =  − 0.7504, *p* = 0.4567).

Completion of the treatments required a considerable number of penultimate and virgin adults of similar age so that we had to use some females multiple times. We ensured that we only re-used females that had no contact with males in previous tests. Males and females were always unrelated, but in some tests, two of the four females were siblings.

### Test arenas

The tests were conducted in test arenas (Fig. 1) simulating semi-natural conditions in which females were randomly distributed and males could move freely. The floor of the arenas was covered with fresh packing paper, on which males could easily walk. The backend of the arena had an air permeable window through which a ventilator produced a slight airflow to ensure pheromone flow within the arena. The starting point for males was at the front end in a central position and consisted of a roughened plastic stick positioned with an inclination of approximately 45°. Each test arena was fitted with the same four applications to fix the frames with the females in identical positions (front, back and two in the middle). In the high mate availability treatment, the four females were randomly positioned on the four spots. In the low mate availability tests, the single adult, virgin female was always located in the front position closest to the male starting point, and the three penultimate females were randomly distributed to the remaining positions.

### Test procedure

Between July 14 and August 16 2016, the experiments took place outside on the roof of the Institute of Zoology of the Universität Hamburg. We monitored the mating tests in up to eight test arenas that were placed underneath a waterproof pavilion roof. In clean and prepared arenas, males were gently placed on the top of the roughened plastic stick (starting point). The time males left the plastic stick was defined as the start of mate searching. Males that did not start their mate search within 45 min were replaced. Males that were caught at the edges of the arena were reintroduced to the starting point and the clock was restarted. Males that ended up in the web of a penultimate female in the low mate availability treatment were given the chance to leave this female for 45 min until they were reintroduced to the starting point. However, males that visited penultimate females might also choose an opportunistic mating strategy, in which they mate with females while they moult to sexual maturity and are defenceless (Uhl et al., 2015). To take this into account, we analysed our data with and without males that had contact to penultimate females. Since the results did not deviate from each other, we only present the analysis with the total sample size and provide the other results as Supplemental Information 2.

During the mating tests, we monitored which female was visited, the duration of copulation and whether the male was cannibalized or not. We also scored whether surviving males copulated a second time with the first mating partner (monopolization) or whether they left the female. We defined that males left the female when there was no contact with the web or frame of the female and a clear absence of male dragline silk attachments for at least one hour. In such cases, we presumed an intention to continue mate searching and follow a bigynous tactic.

### Statistical analysis

We used R (R Developmental Core Team, 2014) for data analyses. We performed parametric tests for normally distributed data and otherwise non-parametric tests.

#### Mate searching and localization

Our predictions rely on the assumption that males estimate the density of adult virgin females via sex pheromone quantity. Thus, mate searching and localization should also be influenced by the number of signalling females. We tested whether latencies until mate search started and the probability of reintroduction after 45 min differed between treatments. Several previous studies from our lab found no evidence for sequential male mate choice in *A. bruennichi* (Schulte, Uhl & Schneider, 2010). We nevertheless explored with binomial tests whether male mate search was directed towards the largest, most attractive female, or to the closest female.

#### Copulation duration and sexual cannibalism

We generated multivariate models to test influences of main effects and covariates on different dependent variables. We simplified the model by removing one variable at a time. Afterwards, we compared each simplified model with the original model using likelihood ratio tests. One by one, we omitted all variables that led to non-significant changes of deviance and removed the least-significant variable first. In the end, we kept the model that successfully explained more variation in the data than the other models.

To test whether copulation duration depended on mate availability, we applied a Cox regression model using the package “survival” (Therneau, 2014). As further explanatory variables, we included female size (tibia-patella length), female condition (residuals of the linear regression between female test weight and female size; see Jakob, Marshall & Uetz, 1996) and the relative weight change of males between adulthood and test day. We used the relative weight change rather than the residual-based male condition that we used for females because earlier studies found a significant effect on male mating decisions (Cory & Schneider, 2018). In a separate Cox regression model with a reduced sample size (we could not measure the legs of four males), we also tested whether male size affected copulation duration.

To test whether mate availability affected the probability of sexual cannibalism, we applied a binary logistic regression. We used the same female and male traits as above.

#### Mating decisions in surviving males

The sample size of surviving males was fairly small, which was why we only conducted a Fisher exact test to explore whether males of the low mate availability treatment would rather stay with the first mating partner than to continue mate searching.

## Results

### Mate searching and localization

In the low mate availability treatment, males started mate searching significantly later than in the high mate availability treatment (high mate availability: median = 32 s, interquartile range = 184 s; low mate availability: median: 266 s, interquartile range = 580 s; Mann–Whitney *U* test: *N* = 50, *W* = 202, *p* = 0.0328). Moreover, we found a tendency that males were more disoriented in the low mate-availability treatments, so that males had to be reintroduced to the starting point after 45 min more frequently than males in the high mate availability treatment (high mate availability: 16%; low mate availability: 56%; Pearson Chi^2^ test: *N* = 50, Chi^2^ = 3.4286, *p* = 0.0641).

In the low mate availability treatment, 7 of 25 males visited a penultimate female first. A prior visit of a penultimate female had no effect on the rate of cannibalism (Fisher exact test: *N* = 25, *p* = 0.6592) or on the duration of copulation (Mann–Whitney *U* test: *N* = 25, *W* = 76, *p* = 0.4584) with the receptive female. In the high mate availability treatment, 48% of males mated with the closest female in the arena, which deviates significantly from a 25% chance (Binomal test: *N* = 25, *p* = 0.0178). We found no evidence that males were more likely to visit the heaviest female (Binomal test: *N* = 25, *p* = 0.4872).

### Copulation duration and cannibalism rate

**Figure 2:**
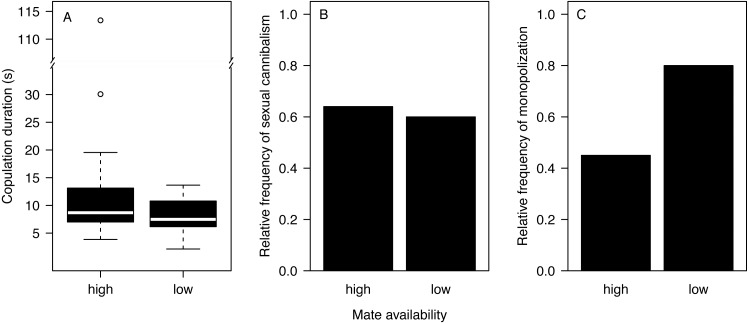
Effects of mate availability on mating behaviour. Effects of mate availability (high, low) on (A) the copulation duration (Cox regression: *N* = 50, Chi^2^ = 3.6061 *p* = 0.0576); (B) the relative frequency of sexual cannibalism (logistic regression: *N* = 50, df-deviance = 0.3082, *p* = 0.5788); and (C) the relative frequency of males that survived the first copulation and copulated a 2nd time with the same female (monopolization) (Fisher exact test: *N* = 19, *p* = 0.1698).

**Figure 3:**
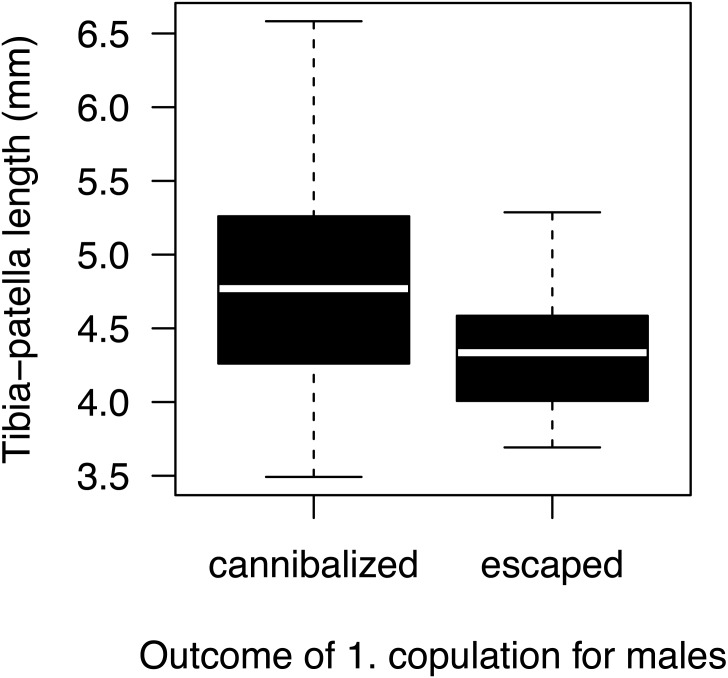
Male size effect on sexual cannibalism. Males that were cannibalized by the female during their first copulation were larger in body size than males that escaped the female attack (logistic regression: *N* = 50, df-deviance = 4.97, *p* = 0.0258).

Most males (90%) copulated less than 15 s (Fig. 2A) and one outlier male from the high mate availability treatment copulated for 113 s. The duration of copulation was not affected by mate availability or any other effect that we tested (Table 1) regardless of whether the outlier was included or not. Therefore, we kept this male in the final analysis. Unlike in previous studies using different populations from the same species (Fromhage, Uhl & Schneider, 2003; Welke & Schneider, 2010), copulation duration was not significantly associated with the probability of sexual cannibalism (Mann–Whitney *U* test: *N* = 49, *W* = 327, *p* = 0.3944). The overall rate of cannibalism was ca. 60% and did not significantly vary with mate availability (Fig. 2B; Table 2). The only significant predictor of sexual cannibalism was a large body size of males (Table 2, Fig. 3).

**Table 1:** Results of the Cox regression model testing effects of mate availability (low, high) on the copulation duration (*N* = 50). The effect of male size was tested in a different model because the sample size was reduced (*N* = 46). The coefficients (coef) and their standard errors (se), and the hazard ratios (exp(coef)) are taken from the full model.

Effect	coef ± se	exp(coef)	Chi^**2**^	*df*	*p*
Availability of virgin ♀♀(low)	0.5743 ± 0.3166	1.7759	3.6061	1	0.0576
♀ size	0.0703 ± 0.1177	1.0728	0.3567	1	0.5503
♀ condition	0.0052 ± 0.0026	1.0052	3.0991	1	0.0783
♂ relative weight change	−2.0859 ± 1.6427	0.1242	1.4882	1	0.2225

Effect	coef ± se	exp(coef)	Chi^**2**^	*df*	*p*
♂ size	0.1032 ± 0.2499	1.1087	0.1683	1	0.6816

**Table 2:** Results of the binary logistic regression testing effects of mate availability (low, high) on the cannibalism rate (*N* = 50). The effect of male size was tested in a different model because the sample size was reduced (*N* = 46). The estimates and standard errors (SE) of the estimates are logit-transformed. Brackets show estimates and standard error of the full model and results of the minimal adequate model are without brackets.

Effect		Estimates ± SE	df-deviance	*df*	*p*
Intercept	(Reference level: high availability)	(−3.0123 ± 2.2115) 0.4895 ± 0.2914			
Availability of virgin ♀♀	(low availability)	(−0.3185 ± 0.6160)	0.3082	1	0.5788
♀ size		(0.4500 ± 0.2861)	2.2874	1	0.1304
♀ condition		(−0.0023 ± 0.0056)	0.1676	1	0.6822
♂ relative weight change		(−2.6694 ± 3.8577.)	0.3953	1	0.5295

Effect		Estimates ± SE	df-deviance	*df*	*p*
Intercept		−5.0451 ± 2.7963			
♂ size		1.2490 ± 0.6213	4.9700	1	0.0258

### Mating decisions in surviving males

A total of 63.2% of the surviving males remated and monopolized the first mating partner. While nearly all males (eight of 10) stayed with the first mating partner in the low mate availability treatment, only four of the nine surviving males monopolized the female under high mate availability. However, the likelihood of monopolization did not significantly differ between treatments (Fig. 2C; Fisher exact test: *N* = 19, *p* = 0.1698), which might be due to the very small sample size.

## Discussion

Theory predicts that mating opportunities should strongly influence mating investment (Kokko & Rankin, 2006; Trivers, 1972). In *A. bruennichi*, males can increase their mating investment by prolonging copulation duration, which also increases the risk of sexual cannibalism. Due to their short mating season (Zimmer, Welke & Schneider, 2012), we expected that males would quickly respond to current information about mate availability. We predicted that under a low availability of adult, virgin females, males would copulate longer and be more often cannibalized than under a high availability of adult, virgin females. Under the latter condition, survival and a bigynous mating tactic would yield the largest fitness return. However, we found no evidence for a strategic adjustment of mating tactic to different levels of mate availability during mate searching.

Contrary to our predictions, neither the probability of sexual cannibalism nor the duration of copulation differed between high or low mate availability. Also, there was no significant evidence that males would leave the first mating partner after the first copulation and presumably engage a bigynous mating tactic when additional adult virgins were nearby. This was unexpected because low mate availability renders a bigynous mating tactic risky and a self-sacrificial strategy with a single, long copulation should be the safer option to enhance male fitness. Even though frequency differences complied with the expected direction, sample size and power were likely too small to statistically verify the effect.

Despite our failure to detect any effect of mate availability on male mating investment, we suggest that variation in female availability is biologically relevant for *A. bruennichi* males. *A. bruennichi* is widespread in Europe and can occupy meadows in high densities (Bruggisser et al., 2012; Krehenwinkel & Tautz, 2013; Kumschick et al., 2011). While the spatial distribution of females may not be a problem, males have to deal with temporal changes in the availability of adult, virgin females (Zimmer, Welke & Schneider, 2012). As in many spider species, the availability of adult, virgin females is very low, especially in the beginning of the mating season, and the protandrous males strongly compete for access to the early maturing females (Elias, Andrade & Kasumovic, 2011). Moreover, the combination of male scramble competition and low mate rejection in females (Schneider et al., 2006) leads to a swift disappearance of virgin females from the mating pool. Thus, the density of adult, virgin females is very dynamic throughout the season, and it should be adaptive for males to integrate social information in their mating decisions. This might be most important for populations in Northern Europe where the mating season is very short. However, in this study, we used spiders derived from Mediterranean populations where seasonal constraints and competition may be less severe. We chose individuals from Southern Europe because unlike in Northern males, their mating decisions are not influenced by female body size (Cory & Schneider, 2018), for which we could not control in our high mate availability treatment. In addition to this difference, Southern males also respond to female cues and shorten their developmental time to the presence of those (Cory & Schneider, 2018).

In contrast to our experiment, most studies tested effects of social experience on mating decisions. Here, individuals received social information (e.g., absence and presence of social cues) for a much longer period of time than in our study. It is well established that social information during development can affect life history traits and mating decisions (Kasumovic & Brooks, 2011; Rodriguez, Rebar & Fowler-Finn, 2013) such as mating preferences (Hebets, 2003; Rutledge, Miller & Uetz, 2010; Stoffer & Uetz, 2016). For instance, it was found that females of the wolf spider *Schizocosa ocreata* were more choosy as adults if they had a high encounter rate of males during development (Stoffer & Uetz, 2015). Hence, mate availability might have a strong impact on mating decisions, but individuals may need enough time to process social information.

We argue that male mating investment will strongly rely on information about adult, virgin female availability, particularly in mating systems where males are adapted to locate receptive females by sex pheromones. To account for this, we manipulated the number of adult, virgin females while leaving the density of webs with females constant. We used penultimate females in the low mate availability treatment because they do not produce the pheromone known for adult, virgin females (Chinta et al., 2010). However, absence and presence of a pheromone and the ability to distinguish do not necessarily mean that males cannot perceive the presence of penultimate females. In the field, males are often found in the vicinity of penultimate females and are known to copulate with them during their final moult (Zimmer & Schneider, 2016). This is beneficial for males because there is no risk of sexual cannibalism from a freshly moulted mate (Uhl et al., 2015). Accordingly, penultimate females do indeed promise future mating opportunities.

Although many studies found that spider males encounter penultimate females (Bel-Venner & Venner, 2006; Biaggio et al., 2016; Erez, Schneider & Lubin, 2005; Johnson, 2005), there is ample evidence that males more easily detect adult virgins than immature or mated females (Gaskett, 2007). In many species, including *A. bruennichi,* males recognize adult, virgin females by sex pheromones that are absent in subadult and mated females (Chinta et al., 2010; Gaskett, 2007; Thomas, 2011). In line with this, behavioural choice tests with *A. bruennichi* confirmed a preference for virgin females (Schneider et al., 2016; Schulte, Uhl & Schneider, 2010). Similar results were found in other spider species (Stoltz, McNeil & Andrade, 2007; Tuni & Berger-Tal, 2012; Watson, 1986). For instance, males of *Stegodyphus lineatus* showed less searching behaviour when presented to immature females than to adult, virgin females (Tuni & Berger-Tal, 2012). Our results agree with this finding. Under the low mate availability treatment, males waited longer until they moved and needed more attempts to find a female compared to males under high mate availability. This suggests that male sensory organs respond to increased quantity of pheromones.

Our low mate availability treatment simulated the beginning of the mating season, in which males could expect future mating chances with adult, virgin females. As the mating season proceeds, future mating chances become increasingly scarce because most females have moulted to adulthood and mated (Zimmer, Welke & Schneider, 2012). Thus, we suggest that future studies should introduce a control treatment with double-mated females instead of penultimate females to simulate conditions late in the season. While females that had copulated once still offer mating opportunities, double-mated females have both copulatory openings plugged and in *A. bruennichi*, copulations into plugged genital openings are unlikely to return paternity gains (Nessler, Uhl & Schneider, 2007). We further suggest a second control-group, which only consists of a single adult, virgin female to test whether males are generally able to assess the presence of females independent of their developmental and mating state.

Another reason for our negative results could be that information about mate availability is not a reliable predictor of fitness, particularly in the absence of information about the degree of male competition. While it seems likely that *A. bruennichi* males can estimate mate availability by female sex pheromones, it is unknown whether males receive cues from other males to make informed decisions. Simultaneous mate choice experiments showed that *A. bruennichi* males do not modify their mate choice decisions in the presence of silk cues from rival males (Schneider et al., 2016). On the other hand, it was found that males decrease the duration of courtship if a rival male is present in the female web (Schneider & Lesmono, 2009). Similarly, males copulated longer and more likely plugged the female in the presence of a rival in the closely related species *Argiope lobata* (Hirt, Ruch & Schneider, 2017). We, therefore, propose that future studies with *A. bruennichi* should integrate information on male density either by testing it as a single factor or in relation to the sex ratio.

Contrary to several previous studies on *A. bruennichi*, we could not confirm the well-established positive relationship between copulation duration and cannibalism risk (Fromhage, Uhl & Schneider, 2003; Nessler, Uhl & Schneider, 2009; Schneider et al., 2006; Schneider & Lesmono, 2009; Welke & Schneider, 2010). We suggest that this interesting difference may be explained by differences in body size between populations. All previous studies used Northern populations, while we used individuals from the Mediterranean area that are larger and more heterogeneous in body size (Cory & Schneider, 2018; Krehenwinkel & Tautz, 2013). Since size differences between sexes matter in sexually cannibalistic spiders (Wilder & Rypstra, 2008), such differences might have affected our results. In contrast to previous studies, we detected size dependent male survival and a benefit of small male size. Observations suggest that larger males were less likely to escape because females were more successful in grasping larger males. A small size advantage of males in avoiding sexual cannibalism was already shown in other studies with spiders (Elgar, Schneider & Herberstein, 2000; Schneider & Elgar, 2001), although the opposite was also found (Elgar & Jones, 2008; Elgar, Schneider & Herberstein, 2000; Persons & Uetz, 2005). Possibly, size related survival differences between males have obscured the relationship between copulation duration and sexual cannibalism.

To our knowledge, this study is the first that tested immediate effects of different levels of mate availability on male investment in a mating system that strongly relies on chemical communication. Although chemical communication is involved in mating interactions of many species, the use of chemical cues is poorly understood (Wyatt, 2014). In comparison, we find many studies on mating systems where males can assess female availability by vision (Barrett, Evans & Gasparini, 2014; Berglund, 1995; Lauer, Sih & Krupa, 1996). For instance, it was found that pipefish males (*Syngnathus typhle*) were only choosy if they were exposed to a high density of females during decision-making (Berglund, 1995). In line with this, a study on social experience in guppies (*Poecilia reticulata*) exposed males visually to a low or high female availability and found that, thereafter, large males showed a higher sexual interest in the current female if other mating opportunities were absent (Barrett, Evans & Gasparini, 2014). In contrast, water strider males (*Aquarius remiges*), that were housed under different density treatments with either females or males, only adjusted their pre-copulatory mating effort if they had experienced a high density of males before (Lauer, Sih & Krupa, 1996). Independent of whether social information was manipulated during or before male mating decisions were tested, this diversity of findings shows that future research should be based on an integrated framework of information about mate perception, mating constraints and ecological conditions.

## Conclusion

Life history theory predicts a trade-off between the current and the future mating effort (Stearns, 2000). Which balance of investment maximizes fitness of males should strongly depend on prospective mating opportunities (Kokko & Rankin, 2006). However, adaptations to adjust investment to mate availability may only evolve if males can gain sufficient information about their social environment. Little is known about information use in a sensory world based on volatile chemicals particularly in the context of mating strategies. Because of their low maximal mating rate, males of sexually cannibalistic spiders are under strong selection to optimize their investment. More studies on such systems may proof useful to further our understanding of chemical information and how it is integrated into decision making. While several studies found that males gain social information through chemical cues by females (Gaskett, 2007; Greenfield, 1981; Holwell, Barry & Herberstein, 2007; Maxwell, Barry & Johns, 2010; Schulz, 2013) it is much less clear whether males can detect chemical cues of rival males. More studies are desired that address whether and how males can perceive rivals and integrate competition in their mating decision. For instance, male spiders may assess other males either by chemical cues or by silk threads that males leave behind during mate search. Besides proximate reasons, ecological and behavioural constraints resulting from the mating system (e.g., mating systems with sexual cannibalism) may also influence adaptations and prevent that males will flexibly respond to mate availability. Here, empirical studies could test whether an interaction between mate availability and risk of sexual cannibalism influences male mating decisions.

##  Supplemental Information

10.7717/peerj.5360/supp-1 Supplemental Information 1 Raw data

10.7717/peerj.5360/supp-2 Supplemental Information 2 Supplemental material

## Additional Information and Declarations

### Data Availability

The following information was supplied regarding data availability: The raw data are provided in a Supplemental File.
