# Supplementary material for: Mate availability does not influence mating strategies in males of the sexually cannibalistic spider Argiope bruennichi
Source: PeerJ. 2018 Aug 7;6:e5360. doi: 10.7717/peerj.5360 (PMC6086085; doi:10.7717/peerj.5360)
Supplement: Supplemental Information 2 [file peerj-06-5360-s002.docx]

**Supplemental material:**

Here, we present the results excluding males that had prior contact with penultimate females:

**Table S1: Results of the Cox regression model testing effects of mate availability (low, high) on the copulation duration (N=43).**

The effect of male size was tested in a different model because the sample size was reduced (N=40).

| **Effect** | **coef** | **exp(coef) ± se** | **Chi^2^** | **df** | **p** |
| --- | --- | --- | --- | --- | --- |
| Availability of virgin ♀♀(low) | 0.6070 | 1.8349 ± 0.3445 | 2.0413 | 1 | 0.1531 |
| ♀ size | 0.1174 | 1.1246 ± 0.1306 | 0.8016 | 1 | 0.3706 |
| ♀ condition | 0.0075 | 1.0075 ± 0.0042 | 2.3084 | 1 | 0.1287 |
| ♂ relative weight change | -2.0092 | 0.1341 ± 1.7564 | 0.9777 | 1 | 0.3228 |
| **Effect** | **coef** | **exp(coef) ± se** | **Chi^2^** | **df** | **p** |
| ♂ size | 0.1220 | 1.1130 ± 0.8852 | 0.1705 | 1 | 0.6796 |

The coefficients (coef) and the exponentiated coefficients (exp(coef)) and their standard errors (se) are taken from the full model.

**Table S2: Results of the binary logistic regression testing effects of mate availability (low, high) on the cannibalism rate (N=43).**

The effect of male size was tested in a different model because the sample size was reduced (N=40).

| **Effect** |  | **Estimates ± SE** | **df-deviance** | **df** | **p** |
| --- | --- | --- | --- | --- | --- |
| Intercept | (Reference level:  high availability) | (-2.9879± 2.3826)  0.4895 ± 0.2914 |  |  |  |
| Availability of virgin ♀♀ | (low availability) | (-0.3838 ± 0.6530) | 0.3402 | 1 | 0.5597 |
| ♀ size |  | (0.4750 ± 0.3088) | 2.5175 | 1 | 0.1126 |
| ♀ condition |  | (0.0003 ± 0.0075) | 0.0019 | 1 | 0.9653 |
| ♂ relative weight change |  | (-0.8524 ± 4.0854) | 0.0437 | 1 | 0.8345 |
| **Effect** |  | **Estimates ± SE** | **df-deviance** | **df** | **p** |
| Intercept |  | -5.4241 ± 3.0503 |  |  |  |
| ♂ size |  | 1.3399 ± 0.6816 | 4.6050 | 1 | 0.0319 |

The estimates and standard errors (SE) of the estimates are logit-transformed. Brackets show estimates and standard error of the full model and results of the minimal adequate model are without brackets.
